# COMPARATIVE STUDY OF DISTAL RADIUS FRACTURE FIXATION WITH A VOLAR PLATE VERSUS KIRSCHNER WIRES

**DOI:** 10.1590/1413-785220263402e291159

**Published:** 2026-05-22

**Authors:** Karina Seabra de Oliveira, Karen Cristina Barbosa Chaves, Paola Augusto Gomes, Gabriel Kosurian de Souza Sayegh, Vitor Augusto Queiroz Mauad, Fernando Nogueira Zambone Pinto, Rafael Saleme Alves, Edson Pereira de Lima, Paulo Cesar Vieira dos Santos

**Affiliations:** 1 Faculdade de Medicina do ABC, Sao Paulo, SP, Brazil.

**Keywords:** Distal radius fracture, Kirschner Wires, Bone plates, Wrist injuries, Fraturas Distais do Rádio, Fios de Kirschner, Placas Ósseas, Traumatismos do Punho

## Abstract

**Introduction:**

Distal radius fractures are the most common fractures of the human skeleton and can be surgically treated using Kirschner wires (K-wires) or a volar plate.

**Objective:**

To analyze and compare the post-surgical results of patients treated with K-wire and volar plate for distal radius fracture.

**Methods:**

Retrospective, quantitative study with medical records and postoperative evaluation questionnaires in ten patients divided into two groups: K-wire and volar plate. The study was conducted at the Hospital de Clínicas Dr. Radamés Nardini, Mauá - SP.

**Results:**

Distal radius fractures were more common in the dominant upper limb, with the volar plate group tending to be older. Volar plate patients had later procedures (median of 13 days), compared to 4 days for K-wire patients. Functional assessment showed better outcomes for the volar plate group, including grip strength. Dorsal flexion loss was 5.2% for K-wire and 21.8% for volar plate when compared to the contralateral limb (P<0.05).

**Conclusion:**

Volar plate fixation showed reduced functional limitations, while K-wire fixation demonstrated a shorter time from trauma to surgery and better range of motion. The small sample size and lack of systematic allocation limit the validity of these results. ***Level of Evidence III; Retrospective comparative study.***

## RESUMO

**Introdução:**

As fraturas do rádio distal são as fraturas mais comuns do esqueleto humano e podem ser tratadas cirurgicamente utilizando fios de Kirschner (K-wires) ou uma placa volar.

**Objetivo:**

Analisar e comparar os resultados pós-cirúrgicos de pacientes tratados com K-wire e placa volar para fratura do rádio distal.

**Métodos:**

Estudo retrospectivo e quantitativo com prontuários médicos e questionários de avaliação pós-operatória em dez pacientes divididos em dois grupos: K-wire e placa volar. O estudo foi realizado no Hospital de Clínicas Dr. Radamés Nardini, Mauá - SP.

**Resultados:**

As fraturas do rádio distal ocorreram principalmente no membro superior dominante, com o grupo da placa volar sendo tendente a ser mais envelhecido. Pacientes com placa volar realizaram o procedimento mais tardiamente (mediana de 13 dias), enquanto no grupo K-wire a mediana foi de 4 dias. A avaliação funcional mostrou melhores resultados para o grupo da placa volar, incluindo força de preensão. A perda de flexão dorsal foi de 5,2% para K-wire e 21,8% para a placa volar quando comparado ao membro contralateral (P<0,05).

**Conclusão:**

A fixação com placa volar apresentou menores limitações funcionais, enquanto a fixação com K-wire demonstrou menor tempo entre trauma e cirurgia e melhor amplitude de movimento. O pequeno tamanho da amostra limita a validade dos resultados. ***Nível de evidência III; Estudo retrospectivo comparativo.***

## INTRODUCTION

Extra-articular distal radius fractures represent a significant challenge in contemporary orthopedic practice, particularly due to their high incidence in elderly populations.^1^ These injuries, often resulting from falls or low-energy trauma, have a considerable impact on patients’ quality of life, with repercussions that extend beyond physical recovery, affecting the functionality of the upper limb and the ability to perform daily activities.^2^ The complexity of treating these fractures arises from factors such as variability in clinical presentation, the presence of comorbidities, and the necessity to restore both the anatomy and function of the wrist.^3^

Surgical treatment has become a common approach for distal radius fractures, especially when closed reduction is not achieved.^4^ In this context, volar plate fixation and the use of Kirschner wires (K-wires) emerge as two of the most discussed options in the orthopedic literature.^5^ According to recent studies, volar plate fixation offers superior stabilization, allowing for faster functional recovery and a lower rate of complications, such as infections and non-union.^6^ This is attributed to the design of the plates, which promotes effective compression of the bone fragments and enables early mobilization of the wrist.^7^

On the other hand, fixation with Kirschner wires, while less invasive and technically simpler, may result in a higher incidence of complications, such as wire migration and the need for additional interventions for removal^8^.

Furthermore, early mobilization is often more limited, which can negatively impact the patient's rehabilitation^9^.

## METHODS

Retrospective, comparative, descriptive and quantitative study based on patients with unilateral distal radius fracture classified like AO 2R3A2 from February 2023 to July 2023 submitted for volar plate or k-wire fixation at Hospital das Clínicas Dr. Rafamés Nardini in Mauá-SP, according to exclusion criteria: non-traumatic fractures; open fractures of the distal radius; injuries with previous sequelae of the upper limb; joint and bilateral fractures; immature skeleton; loss of outpatient follow-up. All the patients signed an Informed Consent Form, and the research was approved by the Local Research Ethics Committee (CAAE: 74031023.8.0000.0082). The epidemiological data, which were descriptively analyzed, included the variables: age, gender, occupation, and the characteristics of both the fracture and the main limb, trauma mechanism, and Δt (trauma - surgery). The immobilization period with a splint is 2 weeks for the volar plate and 6 weeks for the K-wire after surgery. The K-wire and splint were removed simultaneously in the outpatient clinic. Functional, clinical, and radiographic assessments were conducted six months after surgery. Postsurgical assessments were performed to measurements of range of motion, grip strength, volar tilt, radial inclination through the goniometer, upper-limb global grip strength was measured using a dynamometer and functional parameters at least 11 items were evaluated through the quickDASH, a self-reported questionnaire in which the response options are presented as 5-point Likert scales. The quickDASH score calculator ranges from 0% (no disability) to 100% (most severe disability). Statistical significance was established at P<0.05 using the Mann-Whitney test in the GraphPad Prism 9 software.

## RESULTS

Ten patients were included in the analysis, with 5 patients for the volar plate group and 5 for the K wire group. Predominance of distal radius fracture in the dominant upper limb trended towards older age at a median of 61.7 years old for the volar plate group against 55. Both groups consisted exclusively of women. Most of the patients in the K wire group were manual workers (Table 1).

**Table 1:** Comparison between volar plate group vs k-wire group.

	Volar Plate	K wire
Age (interval)	61,7 (21-69) years	55 (34-73) years
Gender (men: women)	02:03	02:03
Occupation	Active labor workers (40%)	Active labor workers (60%)
Energy of trauma	Low (40%)	Low (80%)
Upper limb fracture	Right (80%)	Right (80%)
Dominant side	Right (80%)	Right (100%)
Exposed fracture	No	No
Associated injuries	No	No
Δt trauma-surgery (interval)	13 (3-18) days	4 (2-17) days

Volar plate patients tended to have their procedures later at a median of 13 days, this value was 4 on the K wire group(Table 1). Postsurgical immobilization period with a splint of 2 weeks for the volar plate and 6 weeks for the k-wire. No complications were reported.

Six months after the surgery, all the patients who participated in the research had already completed the rehabilitation protocol under the supervision of a physiotherapist, and manual workers had returned to work. The other patients were retired but had returned to basic daily activities.

The assessment of the upper limb's functionality was through the quickDASH questionnaire, showing better functional outcome for the volar plate group (P=0.135), as well as better grip strength (P=0,151) (Table 2).

**Table 2:** Clinical, radiographic, and functional outcomes.

Clinical Outcomes	Volar plate		K Wire		
	Value Median (min-max)	%	Value Median (min-max)	%	P value
Dorsal flexion	56° (24-70°)	16.7% (5.4-52%)	76° (52-90°)	2.5% (0-12.5%)	0.040
Palmar extension	58° (42-70°)	14.3% (0-31.6%)	52° (50-80°)	23% (11-28%)	0.421
Forearm supination	80° (30-90°)	11.1% (0-67%)	88° (82-90°)	2.2% (0-11%)	0.238
Forearm pronation	80° (70-90°)	9.1% (0-22%)	88° (70-90°)	0 (0-22%)	0.270
Grip strength	24.1 kg (9.8-38.7kg)	21% (1.2-38%)	15.5 kg (5.2-22 kg)	40.1% (14.6-65%)	0.151
**Radiographic Outcomes**					
Volar tilt	10° (2-11°)		11° (10-12°)		0.524
Radial inclination	20mm (2-22mm)		20mm (15-22mm)		1.000
**Functional Outcome**					
quickDASH		2.3% (0-43%)		36.4% (11.4-63.6%)	0.135

In patients of the k-wire group, the median for forearm supination was 88°, whereas in the volar plate group it was 80°. Radiographic outcome showed the median of the volar tilt 11° for the k-wire group against 10° for the volar plate group (Table 2).

On comparison of the targeted variables, there was a significant difference (P<0.05) in the dorsal flexion variability from both techniques, with K wire showing an average loss of 5.2% and volar plate 21.8% when compared to the contralateral member (Table 2).

## DISCUSSION

Distal radius fracture is a pathology that can occur in different age groups, following a bimodal trend, present in both young adults and elderly patients^11–14^. Our sample was slightly older than most literature bases that average around 40-50 years old but reflects the bimodal distribution. This bimodal distribution, usually presented with a slight prevalence of males on the younger quartiles, usually related to car accidents and similar mechanisms while older patients often show a prevalence of low-energy traumas, notabally fall from one's own height ^13,14–17^. Other noteworthy caveats of our group were the expected prevalence of dominant member fractures ^15^ and the absence of related fracture with, in some texts, are present on close to 20% of the cases ^16^. Differences in trauma mechanisms and small sample size are likely to explain these observations.

Regarding postoperative results, a recent study from a University Hospital in Pakistan performed a functional evaluation with a better result in the group with the volar plate approach at 6 months compared to the K-wire. Varshney et al.^18^ obtained a similar conclusion to the present study, with a mean DASH score of 33.47±5.26 in the percutaneous procedure group and 28.03±5^14^. in the volar plate group (p<0.05). However, no significant difference was observed between the two types of treatment at the end of the 12-month follow-up. A meta-analysis also did not record a statistically significant difference in the OR between the comparison of techniques, as observed in the multicenter controlled study in the United Kingdom with a 5-year follow-up^14^There were no differences in wrist extension, pronation, radial deviation, or ulnar deviation during follow-up, which corroborates the present study, in which a statistically significant difference was considered only in wrist dorsiflexion, favoring K-wire (p = 0.040). The 2023 study that analyzed the results of range of motion after 12 months of osteosynthesis with volar plate in 28 patients with AO type B and C fractures observed a result similar to that recorded in the present study degrees in patients treated by this technique with flexion of 60±9 degrees and extension 50±10 degrees, average supination of 77±11 degrees and pronation 84±5 degrees.

The grip strength observed in this study in patients in the postoperative period of osteosynthesis with volar plate corroborates the data found by Albeny et al.^19^ in a retrospective study with 18 patients, recording 24.83 kg with an average loss of 27.9% in relation to the contralateral side. Evaluating functional results, some studies suggest superior grip strength records in the group treated with volar plate compared to treatment with external fixator associated with K wire in the first year after surgery, but at 2 years, there was no difference between the groups^20^.

A published meta-analysis did not observe differences in volar tilt (four trials, 0.1° greater in the K-wire group; 95% CI, −4.6° to 4.9°; p = 0.96). This data is similar to that evaluated in the present study. The data from the study by Choubey et al.^10^ in patients with osteosynthesis with volar plate also converge since at the end of 12 months of observation, there was an average of 17±4 degrees of radial tilt and 7± 5.1 degrees of volar tilt.

## CONCLUSION

The patients evaluated obtained excellent postoperative results in both techniques observed. Despite the small sample size and inherent limitations, we observed that volar locking plates showed better DASH scores and grip strength records at the 6-month follow-up compared with K-wires for distal radius fractures in adults, however, these differences were not statistically significant, while the K-wire technique was superior in recording dorsiflexion range of motion.

Our study reinforces current evidence suggesting that the choice between these two surgical approaches is not trivial and should be based on a careful assessment of the fracture characteristics, the patient's clinical conditions, and treatment objectives. A review of the literature reveals a variety of outcomes and clinical experiences, emphasizing the importance of a personalized approach that considers the specificities of each case. Adequate knowledge of each technique's limitations and strengths, as well as surgeon experience and patient clinical data, are fundamental to sustain a case-by-case decision in the lack of conclusive evidence.

## Data Availability

The data underlying this study are available in the manuscript.
